# Tsetse Salivary Gland Proteins 1 and 2 Are High Affinity Nucleic Acid Binding Proteins with Residual Nuclease Activity

**DOI:** 10.1371/journal.pone.0047233

**Published:** 2012-10-23

**Authors:** Guy Caljon, Karin De Ridder, Benoît Stijlemans, Marc Coosemans, Stefan Magez, Patrick De Baetselier, Jan Van Den Abbeele

**Affiliations:** 1 Department of Biomedical Sciences, Unit of Veterinary Protozoology, Institute of Tropical Medicine Antwerp (ITM), Antwerp, Belgium; 2 Unit of Cellular and Molecular Immunology, Vrije Universiteit Brussel (VUB), Brussels, Belgium; 3 Laboratory of Myeloid Cell Immunology, VIB, Brussels, Belgium; 4 Department of Biomedical Sciences, Unit of Medical Entomology, Institute of Tropical Medicine Antwerp (ITM), Antwerp, Belgium; Metabiota, United States of America

## Abstract

Analysis of the tsetse fly salivary gland EST database revealed the presence of a highly enriched cluster of putative endonuclease genes, including *tsal1* and *tsal2*. Tsal proteins are the major components of tsetse fly (*G. morsitans morsitans*) saliva where they are present as monomers as well as high molecular weight complexes with other saliva proteins. We demonstrate that the recombinant tsetse salivary gland proteins 1&2 (Tsal1&2) display DNA/RNA non-specific, high affinity nucleic acid binding with K_D_ values in the low nanomolar range and a non-exclusive preference for duplex. These Tsal proteins exert only a residual nuclease activity with a preference for dsDNA in a broad pH range. Knockdown of Tsal expression by *in vivo* RNA interference in the tsetse fly revealed a partially impaired blood digestion phenotype as evidenced by higher gut nucleic acid, hematin and protein contents.

## Introduction

Tsetse flies (*Glossina* sp.) are obligate blood feeding insects that transmit protozoan parasites (*Trypanosoma* sp.), the etiological agents of African trypanosomiasis. During the probing and blood feeding interaction, tsetse flies inoculate a complex mixture of salivary components from which the composition has been explored by proteome and transcriptome analyses as well as by functional genomics approaches [1], [2], [3], [4], [5], [6], [7]. It has already been established that tsetse fly saliva interferes with host hemostatic reactions that are initiated at the blood feeding site [3], [8], [9]. Anti-coagulant and anti-thrombotic compounds include respectively the highly potent tsetse thrombin inhibitor [TTI, [8]] and a recently identified apyrase with fibrinogen receptor antagonistic features [3]. Beside interfering with host hemostasis, saliva is highly immunogenic/allergenic in nature [2], [10], [11] and was documented to modulate host inflammatory reactions against the trypanosome, thereby promoting infection onset [12]. This implies that salivary compounds not only contribute to the feeding event but also can be considered as vector-derived virulence factors.

Strikingly, the salivary proteome of *Glossina morsitans morsitans*, a major vector for *Trypanosoma brucei rhodesiense* infections, is dominated by a highly immunogenic 43–45 kDa protein family with a yet unknown function [5], [6]. Initial transcriptome analyses in *G. m. morsitans* revealed that there are at least three homologous genes that contribute to this protein fraction that includes tsetse salivary gland protein 1 (Tsal1) and two isoforms of Tsal2 (Tsal2A and Tsal2B) [6]. A recent, detailed expressed sequence tag (EST) transcriptome analysis described an even higher level of complexity where gene duplication events would have resulted in a minimum of eight genes encoding for this Tsal protein family [7]. Phylogenetic reconstruction based on the assembled EST sequences are supportive for three clades that are represented by (*i*) a set of truncated tsal1-like ESTs, (*ii*) *tsal1* and three other full length ESTs with >90% identity with *tsal1* and (*iii*) four ESTs that encode the closely related *tsal2a* and *tsal2b* [7]. RT-PCR and EST sequencing on different tissues (salivary gland, midgut and fat body) revealed that these genes are nearly exclusively expressed in the salivary gland tissue, with *tsal1* accounting for nearly 15% of all 20,000 sequenced salivary gland ESTs [7]. Also immune screening of a λgt11 salivary gland cDNA expression library with anti-tsetse saliva serum from a tsetse-exposed host, revealed an extremely biased recovery frequency (81.8%) for Tsal2 encoding genes [6]. Here we describe that the encoded tsetse fly Tsal proteins share a significant degree of homology with sugar non-specific endonucleases that can be found in prokaryotic as well as eukaryotic organisms.

The presence of salivary gland transcripts encoding putative secreted nucleases is a recently emerging observation in several blood feeding arthropods including sandflies *Lutzomyia longipalpis* (GenBank accession No.: AY455916.1) and *Phlebotomus argentipes* (SP11, GenBank accession No.: DQ136157.1) [13] and Culex mosquitoes. Recently, actual endonuclease activity was documented in the saliva of the mosquito *Culex pipiens quinquefasciatus* while the functional relevance remains to be elucidated [14]. The responsible protein, CuquEndo, has a predicted molecular weight of 39.3 kDa and exerts double stranded DNA (dsDNA) specific endonuclease activity in an alkaline pH range (pH 7.5–8.5). Calvo *et al*. propose that, given the exclusive expression of CuquEndo in female mosquitoes, the activity is required for the blood feeding process through reducing the blood meal viscosity [14]. However, similar experiments in other mosquitoes, such as *Aedes aegypti* and *Anopheles gambiae*, could not detect functional nucleases in their saliva [14].

In this study, we report that tsetse fly saliva exerts DNA-binding and low rate nuclease activity and we identify the two major tsetse salivary proteins 1 and 2 (Tsal1, 45.6 kDa and Tsal2, 43.9 kDa) as high affinity, DNA/RNA non-specific nucleic acid binding proteins. Unlike the *Culex* endonuclease, the Tsal proteins display only a residual nuclease activity. *In vivo* silencing of Tsal expression through RNA interference suggested that these proteins support blood meal digestion.

## Materials and Methods

### Ethics Statement

The experiments, maintenance and care of mice and rabbits complied with the guidelines of the European Convention for the Protection of Vertebrate Animals used for Experimental and other Scientific Purposes (CETS n° 123). Rodent care and experimental procedures were performed under approval from the Animal Ethical Committee of the Institute of Tropical Medicine (Permit Nrs. PAR013-MC-M-Tryp and PAR014-MC-K-Tryp). Breeding and experimental work with tsetse flies was approved by the Scientific Institute Public Health department Biosafety and Biotechnology (SBB 219.2007/1410).

**Figure 1:**
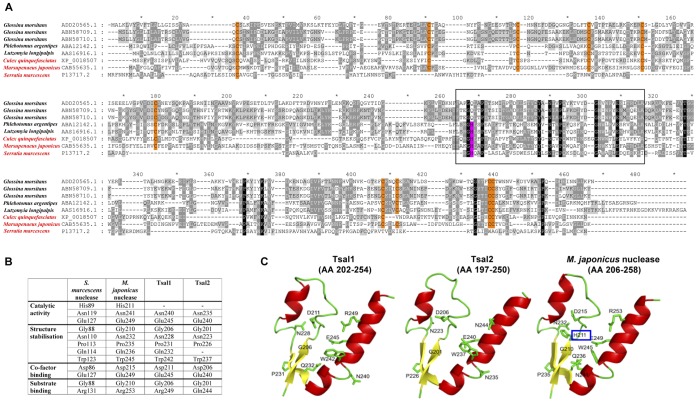
*In silico* analysis of Tsal1 and Tsal2. (**A**) Sequence alignment of Tsal1, Tsal2A and Tsal2B (GenBank accession Nos.: ADD20565, ABN58709, ABN58710) with homologous genes annotated as putative salivary gland nucleases in *Culex quinquefasciatus* (GenBank accession No.: XP_001859795), *Phlebotomus argentipes* and *Lutzomyia longipalpis* sand flies (GenBank accession Nos.: ABA12142, AAS16916), the *Marsupenaeus japonicus* shrimp hepatopancreatic nuclease (GenBank accession No.: CAB55635) and the archetypical *Serratia marcescens* nuclease (GenBank accession No.: P13717). Marking of the species names in red indicates that the nuclease activity for these genes has been confirmed. Cysteine residues are indicated on an orange background. The box delineates the putative nuclease active site region. The residue indicated on a purple background corresponds to the histidine anticipated to be crucial for the catalytic activity of the nuclease. (**B**) Amino acids predicted to be involved in catalytic activity, structure stabilization and co-factor and substrate binding of the *S. marcescens* and the *M. japonicus* nuclease with the homologous residues in Tsal1 and Tsal2. (**C**) Structure prediction of the putative active site regions within the NUC domain of Tsal1 (AA 202–254) and Tsal2 (AA 197–250) and comparison with the predicted structure of the shrimp nuclease active site region (AA 206–258). A histidine residue (boxed) considered important as general base in the catalytic mechanism, is lacking in Tsal1 and Tsal2.

### Tsetse Flies, Saliva Isolates and Salivary Protein Fractions

Tsetse flies *(Glossina morsitans morsitans)* were available from the insectaria at the Institute of Tropical Medicine Antwerp. Saliva was harvested from tsetse flies by dissection as described earlier [15] or by induced probing using a method communicated to us by Dr. Ted Hall (scientist formerly based at Walter Reed Army Institute of Research, Maryland, USA).

**Figure 2:**
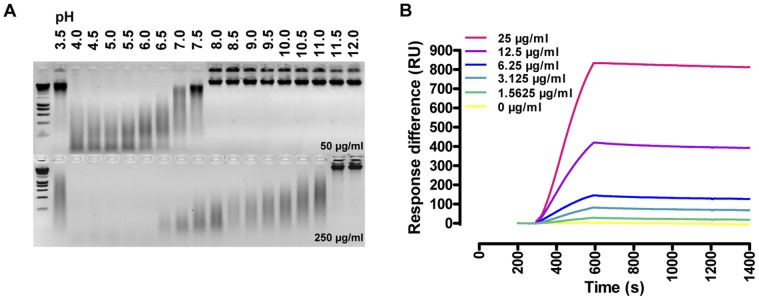
DNA hydrolytic and binding properties of total tsetse fly saliva. (**A**) dsDNase activity in different pH conditions (pH 3.0–12.0, 1 mM Ca^2+^/Mg^2+^) of 50 µg/ml (upper panel) and 250 µg/ml saliva (lower panel) using 50 µg/ml calf thymus DNA as a substrate and analyzed on a 1% agarose gel after 16 h incubation at 37°C; (**B**) Surface Plasmon Resonance (SPR)-based binding experiment with different saliva concentrations (1∶2 dilution series from 25 to 1.5625 µg/ml, pH 4.0) conducted at 30 µl/min onto 300 RU biotinylated dsDNA immobilized on an SA sensor chip.

Briefly, tsetse flies were placed in clean cages that were covered with a glass plate and a heating lid set at 40°C. Flies were allowed to probe onto the heated plate for 30 minutes, followed by scraping the saliva from the glass using a microtome blade. Saliva was resuspended in saline and non-solubilized material removed by a 2 minute centrifugation at 12000×g. Salivary protein fractions were obtained by size exclusion chromatography on a Superdex 200 column connected to an Äkta Explorer (GE Healthcare). Protein concentrations were determined by the BCA kit (Pierce Biotechnology) and aliquots stored at −20°C.

**Figure 3:**
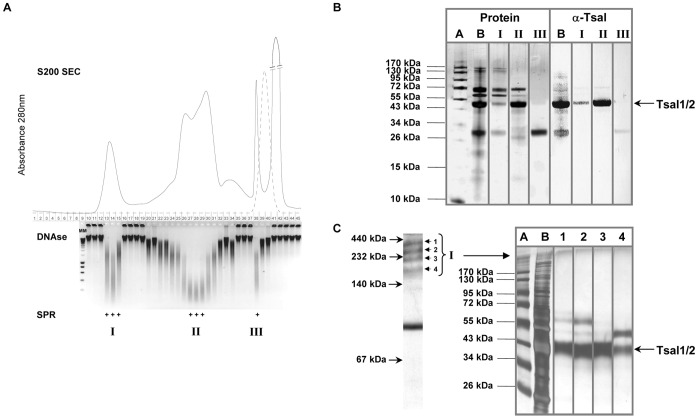
DNA hydrolytic and binding activity in fractions obtained from total tsetse fly saliva. (**A**) Superdex 200 size exclusion chromatogram with indication of the saliva fractions that were individually analyzed for dsDNAse activity (fractions were 1∶5 diluted in reaction buffer to yield final concentrations of 50 mM HEPES pH 7.0, 1 mM Ca^2+^/Mg^2+^ and 50 µg/ml calf thymus DNA). gDNA integrity was assessed on a 1% agarose gel after 16 h incubation at 37°C. 1∶25 diluted fractions were assessed by SPR for binding at pH 4.0 onto 300 RU biotinylated dsDNA immobilized onto an SA sensor chip. Positive peaks (I, II and III) are indicated underneath the agarose gel. The dashed line represents the conductivity profile. (**B**) Silver stained protein profiles of S200 fractions I, II and III [molecular marker (lane A), total saliva (lane B), and lanes I to III corresponding with the protein peaks I, II and III] and western blot analysis using purified rabbit anti-Tsal1&2 polyclonal IgGs. (**C**) Coomassie stained tsetse fly salivary proteins separated under native conditions. The four high molecular weight (HMW) bands (>140 kDa), corresponding to the positive S200 fraction I, were subjected to protein electro-elution, separation under reducing conditions by 10% SDS-PAGE and silver-staining [molecular marker (lane A), total saliva (lane B), and lanes 1 to 4 corresponding with the protein bands 1 to 4]. The 43–45 kDa protein band represents Tsal1/2.

### cDNA Sequence Analysis and Structure Prediction

Full-length cDNA sequences encoding Tsal1 and Tsal2A were picked up from a λgt11 salivary gland expression library [6]. Translated sequences were subjected to NCBI protein-protein BLAST (BLOSUM62 matrix). Alignments were made using the CLUSTALW program and imported into GeneDoc (www.psc.edu/biomed/genedoc). Homology detection and structure prediction was based on comparison of Hidden Markov Models (http://toolkit.tuebingen.mpg.de/hhpred). Generated PDB-files were used to produce 3D-figures with PyMOL (http://pymol.sourceforge.net).

**Figure 4:**
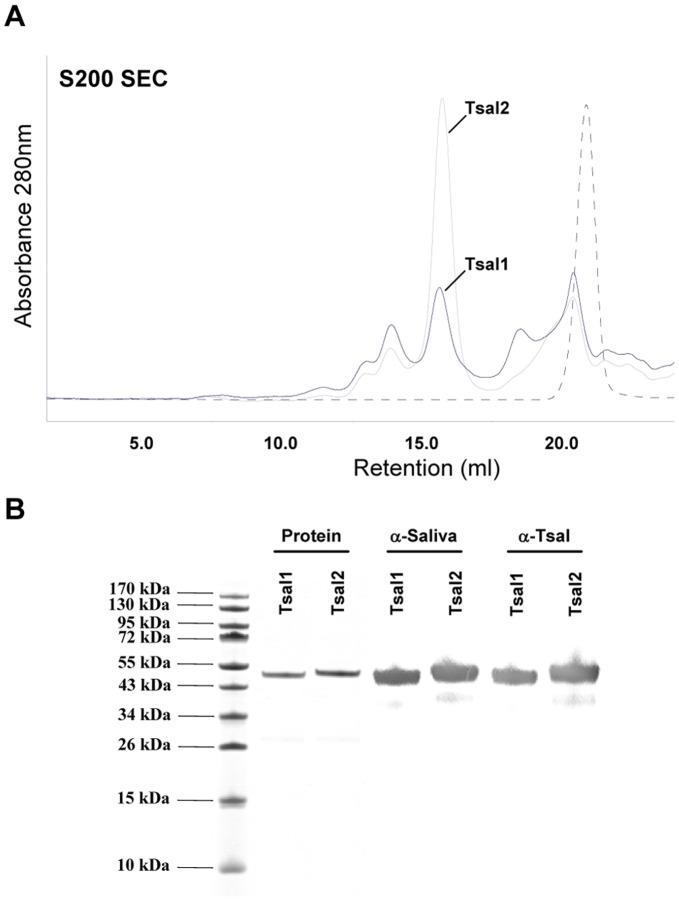
Purification of *E. coli* recombinant Tsal1 and Tsal2. (**A**) Superdex 200 size exclusion chromatogram with indication of the Tsal1&2 protein peaks. The dashed line represents the conductivity profile. (**B**) Coomassie stained protein profile of peak fractions separated on a 12% SDS-PAGE and identity confirmation by western blot analysis using purified rabbit anti-saliva and anti-Tsal IgGs.

**Figure 5:**
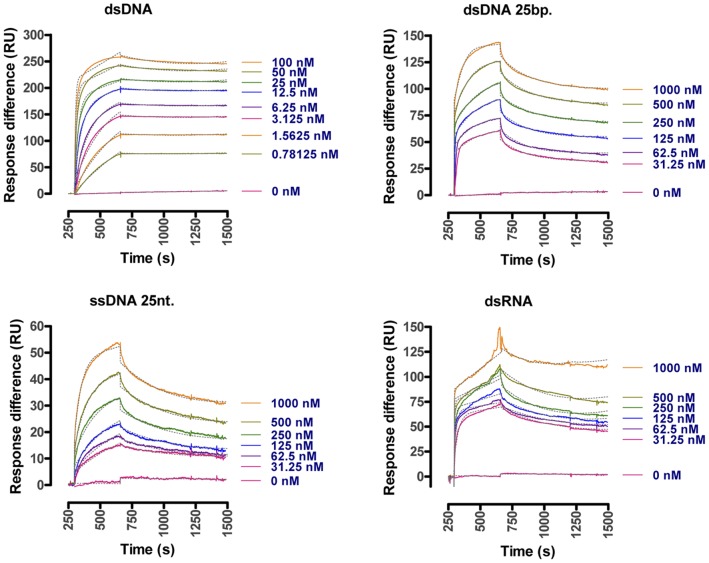
Recombinant Tsal1&2 nucleic acid binding properties. Representative SPR sensograms obtained for binding at 30 µl/min of different nucleic acid analytes (161 bp dsDNA, 25 bp A/T/G/C-scrambled dsDNA, 25 nt A/T/G/C-scrambled ssDNA, 121 bp dsRNA) at the pH optimum of 4.0 onto the same CM5-chip with 1000 RU Tsal1 and Tsal2 coated in respectively flow cells 2 and 4. Flow cell surface regeneration was obtained with 5 M LiCl. Dotted lines correspond to the calculated curves that were obtained by fitting the experimental SPR sensograms for a Langmuir 1∶1 binding model and local R_max_ using the BIA-evaluation software version 4.1.

**Figure 6:**
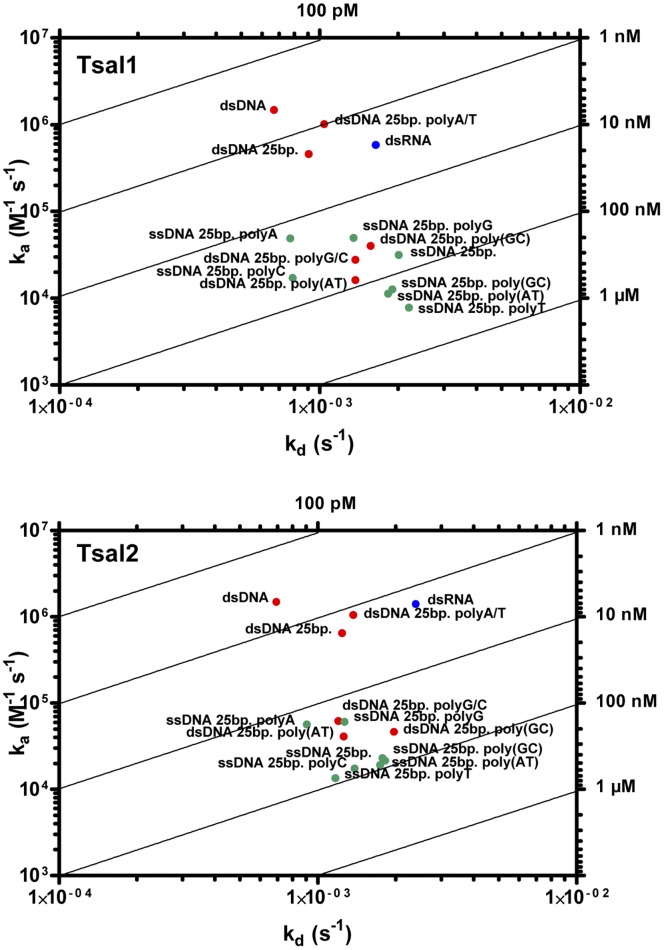
Recombinant Tsal1&2 nucleic acid binding properties. Rate plane with Isoaffinity Diagonals (RaPID) plot of the different nucleic acid (dsDNA, ssDNA, dsRNA) analytes. The kinetic rate values k_a_ (M^−1^s^−1^) and k_d_ (s^−1^) for an analyte to Tsal1&2 at pH 4.0 are plotted onto a two-dimensional diagram with isoaffinity diagonals (K_D_ = k_d_/k_a_). Affinities were determined by fitting the SPR sensograms from different analyte concentrations (1∶2 serial dilution from 1000 to 31.25 nM for the short dsDNA analytes, ssDNA and dsRNA or a 1∶2 dilution series from 100 to 0.78 nM for the 161 bp dsDNA analyte) for a Langmuir 1∶1 binding model and local R_max_ using the BIA-evaluation software version 4.1.

**Table 1:** Tsal-binding kinetic parameters.

	Tsal1			Tsal2A		
	k_a_ (M^−1^s^−1^)	k_d_ (s^−1^)	K_D_ (nM)	k_a_ (M^−1^s^−1^)	k_d_ (s^−1^)	K_D_ (nM)
**dsDNA**	1.5E+06	6.7E−04	0.45	1.5E+06	6.9E−04	0.46
**dsDNA 25bp.**	4.6E+05	9.1E−04	2.0	6.5E+05	1.2E−03	1.9
**dsDNA 25bp. polyA/T**	1.0E+06	1.0E−03	1.0	1.1E+06	1.4E−03	1.3
**dsDNA 25bp. polyG/C**	2.8E+04	1.4E−03	49	6.2E+04	1.2E−03	19
**dsDNA 25bp. poly(AT)**	1.6E+04	1.4E−03	85	4.1E+04	1.3E−03	31
**dsDNA 25bp. poly(GC)**	4.0E+04	1.6E−03	39	4.7E+04	2.0E−03	42
**dsRNA**	5.8E+05	1.6E−03	2.8	1.4E+06	2.4E−03	1.7
**ssDNA 25nt.**	3.2E+04	2.0E−03	64	2.2E+04	1.8E−03	85
**ssDNA 25nt. polyA**	4.9E+04	7.7E−04	16	5.6E+04	9.1E−04	16
**ssDNA 25nt. polyT**	7.8E+03	2.2E−03	281	1.4E+04	1.2E−03	87
**ssDNA 25nt. polyG**	5.0E+04	1.4E−03	27	6.0E+04	1.3E−03	21
**ssDNA 25nt. polyC**	1.7E+04	7.9E−04	46	1.8E+04	1.4E−03	79
**ssDNA 25nt. poly(AT)**	1.1E+04	1.8E−03	162	1.9E+04	1.7E−03	91
**ssDNA 25nt. poly(GC)**	1.3E+04	1.9E−03	151	2.3E+04	1.8E−03	77

Tsal-binding kinetic parameters (k_a_, k_d_ and K_D_) for the different tested nucleic acid analytes determined using surface plasmon resonance with 1000 RU recombinant Tsal1 and Tsal2 immobilized onto a CM5 sensor chip. Sensograms were fitted for a Langmuir 1∶1 binding model with local R_max_ using the BIA-evaluation software version 4.1.

### Cloning, Expression and Purification of Recombinant Tsal1 and Tsal2A

For expression of soluble Tsal1 and Tsal2A in the periplasm of *E. coli*, *tsal1* and *tsal2A* devoid of the natural signal sequence were cloned from the available *Glossina morsitans morsitans* cDNA library into the pET22b plasmid (Novagen) that includes a pelB leader sequence. *Tsal1* was first cloned (sense primer: 5′-GCGCCCATGGATTGTTCGTTAAAAATACCAG-3′/antisense primer: 5′-GCGCGGATCCATTAAATTTTAACAAATTATTAATTTC-3′) into pQE60 (Qiagen) as described previously [11] using the restriction sites *Nco*I and *BamH*I and subsequently subcloned into pET22b using the *Nco*I and *Hind*III sites. *Tsal2a* cloning (sense primer: 5′-CGGCCATGGATCAATGTTCCATTAACATACC-3′/antisense primer: 5′-GATCGGGATCCGAATATTTTAAAAGGCCTTTGACATG-3′) was achieved using the *BamH*I and *Xho*I restriction sites. BL21 cells (Invitrogen) were transformed with pET22b:*tsal1* or pET22b:*tsal2A* and protein expression was induced with 1 mM IPTG in overnight cultures for 3 hours at 37°C. Proteins were purified from periplasmic extracts using Ni-NTA superflow affinity chromatography (Qiagen). Elutes were concentrated on 30 kDa MWCO Vivaspin concentrators (Vivascience) and further purified on a Superdex 200 gelfiltration column connected to an Äkta Explorer (GE Healthcare). Individual fractions were analyzed for activity and purity on 4–12% Bis-Tris gradient gels (Invitrogen). Aliquots were stored at −20°C till further use.

### Generation of Polyclonal IgGs Against Saliva, Tsal1 and Tsal2

Tsal1 and Tsal2A proteins were purified from inclusion bodies as described earlier [11] and were used to immunize New Zealand white rabbits. Animals were immunized three times with 100 µg protein, by priming in Freund’s complete adjuvant and boosting twice in Freund’s incomplete adjuvant. The polyclonal sera were collected 2 weeks after the final booster. Anti-*Glossina morsitans* saliva immune serum was available from a previous study [6]. Pre-immune IgGs and polyclonal IgGs against Tsal1, Tsal2 and total tsetse fly saliva were purified by affinity chromatography using protein G sepharose (GE Healthcare).

**Figure 7:**
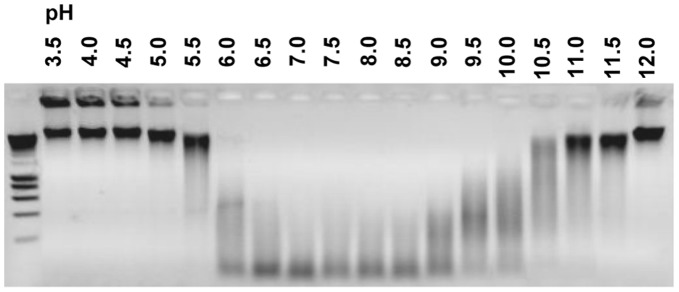
Recombinant Tsal1&2 nuclease properties. Representative qualitative analysis of the nuclease activity exerted by 25 µg/ml recombinant Tsal1 and Tsal2 (results shown for Tsal1) using 50 µg/ml calf thymus DNA as a substrate. gDNA integrity was analyzed by agarose gel electrophoresis after 16 h incubation at 37°C under different pH conditions (pH 3.0 to 12.0) with 1 mM Ca^2+^/Mg^2+^.

### Nuclease Activity Measurement

Nuclease pH optima of total saliva, individual saliva fractions and recombinant Tsal1 and Tsal2 were determined in buffer conditions ranging from pH 3.0 to 12.0. The used buffers were sodium acetate (pH 3.0 to 5.4), MES (pH 5.6 to 6.6), HEPES (pH 6.8 to 7.8), Tris (pH 8.0 to 9.0) and piperazin (pH 9.0 to 12.0) and with 15 mM NaCl. Reactions were performed in the presence of 1 mM CaCl_2_ and MgCl_2_. To determine the cofactor preference, activity was monitored in the presence of a range of divalent ions (Ca^2+^, Mg^2+^, Co^2+^, Cd^2+^, Cu^2+^, Zn^2+^ and Ni^2+^ with chloride as counterions) at 1 mM final concentration and in combination with 0.1 mM EDTA and EGTA. DNA samples were loaded after different incubation times on 1% agarose gels with TBE as running buffer. Substrate specificity was assessed using calf thymus genomic DNA (gDNA, purchased from Sigma), plasmid DNA (pDNA; pcDNA3.1, Invitrogen) purified using the EndoFree plasmid giga kit (Qiagen), in-house produced GFP double stranded RNA [dsRNA, produced using the Megascript RNAi kit (Ambion); see below] and a synthetic single stranded DNA (ssDNA) molecule (Sigma).

**Figure 8:**
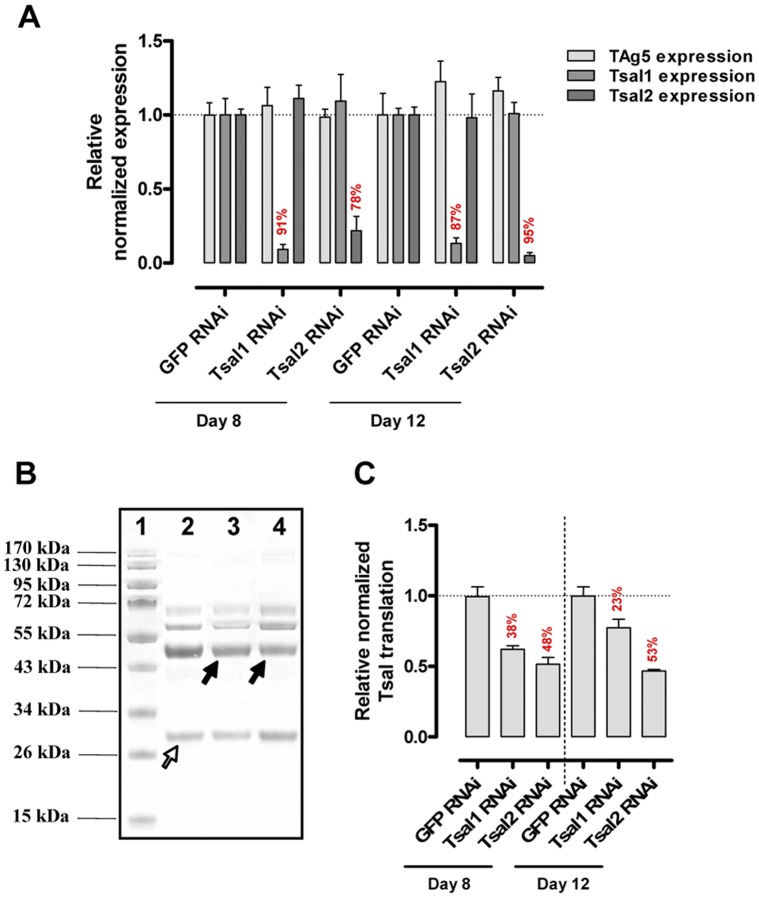
Tsal1 and Tsal2 specific RNAi. (**A**) Relative normalized *tag5, tsal1* and *tsal2* mRNA levels determined using *β-tubulin, tag5* and *gapdh* as reference genes for 5 pools of 3 glands pairs at day 8 and 12 after intrathoracal injection of 15 µg gene-specific or control (GFP) dsRNA (**B**) Example of coomassie-stained protein profiles of saliva harvested from pools of 3 GFP RNAi (lane 2) and Tsal1 and Tsal2 RNAi treated flies at 12 days p.i. (lanes 3 and 4, filled arrows). (**C**) The bar chart represents the specific protein silencing efficiency normalized against the TAg5 protein band (empty arrow, panel B) determined for 5 pools of 3 glands pairs per time point and experimental group. The presented RNAi data are representative for at least 5 independent experiments with 10–30 flies.

The nuclease activity of total saliva and recombinant proteins was quantified by hyperchromicity [16], [17] in flat bottom UV-star 96 well plates (Greiner bio-one) by incubation of 25, 50 and 100 µg/ml calf thymus DNA in 50 mM sodium acetate pH 4.0 and 50 mM HEPES pH 7.0 supplemented with 1 mM CaCl_2_ and 1 mM MgCl_2_. Reactions at pH 4.0 were performed at a physiological 150 mM NaCl concentration. As we found that salts were inhibiting the nuclease activity in the neutral/alkaline pH range, enzymatic activity at pH 7.0 was determined in 15 mM NaCl conditions. Plates were covered with Saran wrap to avoid evaporation. The optical densities (O.D.) were monitored at a 260 nm wavelength for 16 h at 37°C in an Infinite M200 spectrophotometer (TECAN) and recorded using the Magellan 6.0 software. The linear phase in the hyperchromicity plots (ΔO.D._260 nm_ for a 1 cm path length) was used to calculate the specific activity in ΔmAU_260 nm_/min×mg [16], [17]. The gDNA integrity was also analyzed by agarose gel electrophoresis.

### Nucleic Acid Binding Affinity Measurement by Surface Plasmon Resonance (SPR)

General binding characteristics of total saliva were tested by surface plasmon resonance on a BIAcore 3000 system (GE Healthcare), using 300 response units (RU) DNA-biotin [a 186 bp fragment from the Ss-LrpB operator/promoter region of *Sulfolobus solfataricus* [18]] immobilized through the standard procedure onto an activated streptavidine chip (Sensor chip SA, GE Healthcare). Saliva was run under different buffer conditions (pH 4.0, 5.0, 6.0, 7.0 and 8.0, 150 mM NaCl) over the flow cell at different concentrations (1∶2 serial dilution from 25 to 1.56 µg/ml) and a 30 µl/min flow rate. Individual saliva fractions, obtained by Superdex 200 HR10/30 gel filtration (GE Healthcare), were 1∶25 diluted in 50 mM sodium acetate pH 4.0 150 mM NaCl and tested for DNA binding activity using the same DNA-coated sensor chip. The chip was regenerated by an injection of 30 µl 5 M LiCl over the flow cell. In another setup, 3500 RU saliva was immobilized onto a CM5-chip, and 50 nM pDNA was used as an analyte under physiological conditions.

**Figure 9:**
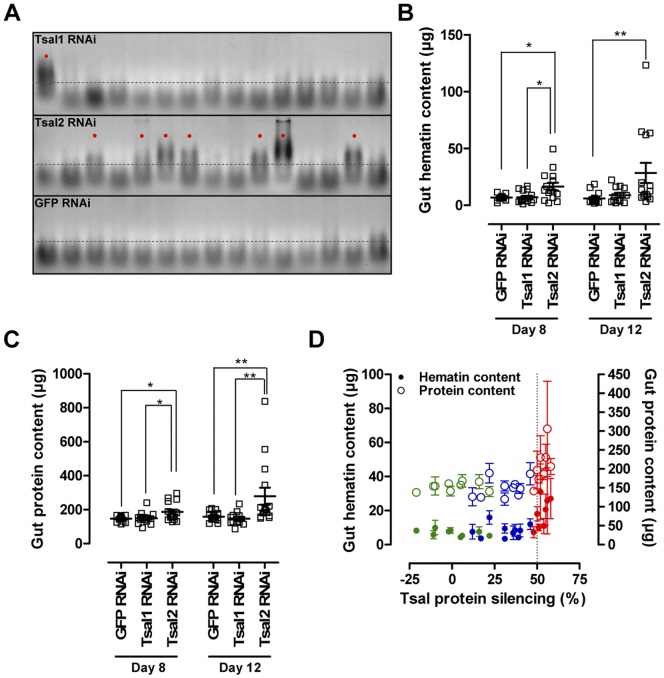
Effect of Tsal1 and Tsal2 specific RNAi on the tsetse fly blood meal digestion physiology. (**A**) Gut homogenates of individual flies after 72 h starvation were run on a 2% agarose gel, allowing the visualization with ethidium bromide of nucleic acid degradation in the digested blood. Presented data in this panel are those for flies at 8 days p.i. A threshold of 300 bp (dotted line) was set to distinguish between the profiles of a perturbed (indicated with dots) and normal digestion. RNAi induced alterations in (**B**) the gut hematin digestion and (**C**) remainder gut protein contents 72 hours after the last blood meal. (**D**) Illustration of the RNAi-mediated effects on gut hematin (•, left Y-axis) and protein contents (○, right Y-axis) in function of protein silencing efficiency (Tsal1 RNAi: blue; Tsal2 RNAi: red, GFP RNAi: green). This graph represents the percentage Tsal protein reduction in the saliva harvested from 3 gland pairs (X-axis) and the average of the individually determined hematin and protein contents in the guts of the 3 corresponding flies. Phenotypic effects of Tsal2 RNAi were confirmed in 3 independent experiments. Statistical analyses were performed using the Mann Whitney test in the GraphPad Prism 5.0 software package; * and ** indicate respectively *P*<0.05 and *P*<0.01.

To determine the affinities of recombinant Tsal1 and Tsal2A for different nucleic acid analytes, 1000 RU of either Tsal1 or Tsal2A was coupled onto a CM5 chip (BIAcore) via amine groups according to the manufacturer’s descriptions. EDC and NHS were used as cross-linking agents and ethanolamine to block free esters. The used analytes tested in the SPR analysis were a 161 bp dsDNA molecule with opposing 20 bp T7 promotors, the corresponding dsRNA, 25 bp ssRNA molecules [polyA, polyT, polyG, polyC, poly(GC), poly(AT)] and 25 bp dsDNA molecules [polyA/T, polyG/C, poly(AT), poly(GC) and a mixed A/T/G/C dsDNA fragment (5′-CACTAGGTCTGATCAGGCAACGTTG-3′)]. For the affinity determination, nucleic acid concentrations ranging from 1000 to 0.78 nM were added to the Tsal-coated chip at a flow-rate of 30 µl/min in a sodium acetate buffer [50 mM sodium acetate (pH 4.0), 150 mM NaCl, 3.5 mM EDTA, 0.005% (v/v) Tween-20)]. Chip regeneration was achieved with 5 M LiCl. Sensograms were fitted for a Langmuir 1∶1 binding model with local R_max_ using the BIA-evaluation software version 4.1 (BIAcore), resulting in association and dissociation constants (k_a_ and k_d_) as output from which affinity (K_D_) values were calculated. χ^2^ values and residuals were analyzed for accuracy of the fitting.

### In vivo RNA Interference (RNAi)

For the *in vivo* functional analysis of Tsal1 and Tsal2A, the RNAi method was applied by the injection of double stranded RNA (dsRNA) molecules with sizes ranging from 400 to 600 bp. The efficiencies of three different dsRNA molecules per transcript were analyzed. As a negative control, GFP targeting dsRNA was generated. Templates for dsRNA production by *in vitro* transcription (IT) were generated by PCR, incorporating opposing T7 promotors (5′-GCTAATACGACTCACTATAGGGAGA-3′) at both ends of the amplicon. To generate the IT-templates for the production of the control dsRNA (GFP dsRNA: 494 bp) and the most efficient gene-specific dsRNAs (Tsal1 dsRNA: 433 bp, Tsal2A dsRNA: 435 bp), plasmids containing the GFP, Tsal1 and Tsal2A full length coding sequences (pCM66:*gfp*, pIZ/V5His:*tsal1*, pFAST:*tsal2a*) were used as PCR templates in combination with following primers: Tsal1(IT) sense (5′-GCTAATACGACTCACTATAGGGAGAACATTGGCCTCTTCGCATTG-3′), Tsal1(IT) antisense (5′-GCTAATACGACTCACTATAGGGAGAACGGTAAGCCACTTGTGGAT-3′), Tsal2A(IT) sense (5′-GCTAATACGACTCACTATAGGGAGATGCCAGCAGATTGTGTAACC-3′), Tsal2A(IT) antisense (5′-GCTAATACGACTCACTATAGGGAGAGTCAGCTAACACCAGACACTTC-3′), GFP(IT) sense (5′-GCTAATACGACTCACTATAGGGAGATGGCCAACACTTGTCACTAC-3′) and GFP(IT) antisense (5′-GCTAATACGACTCACTATAGGGAGAAGAAGGACCATGTGGTCTCT-3′). PCR amplicons were column purified (Qiagen), eluted in nuclease-free water (Ambion) and analyzed on a 1% agarose gel.

The IT was performed using the Megascript RNAi kit (Ambion), following the manufacturer’s instructions. DsRNA was treated with ssRNase and DNase I, followed by column purification, elution in 2×100 µl prewarmed (95°C) nuclease-free water and concentration in a speed-vac (DNA Speedvac, Savant).

For injection purposes, tsetse flies (48 h after the last blood meal) were briefly anaesthetised by cold shock and were injected with a single dose of 15 µg dsRNA. Intrathoracal injections were performed under a binocular microscope, using a 5 µl Hamilton 75RN microsyringe with gauge 30 electrotapered needles.

### Tsetse Fly Feeding Efficiency and Survival

For each RNAi group, at least 30 tsetse flies were individualized in numbered cages. At days 5 and 9 after dsRNA injection, individual flies were fed on anesthetized NMRI mice. In order to quantify the blood meal weights, individual fly weights were measured before and after blood feeding using an analytical balance (Sartorius).

### Salivary RNA Extraction and Quantitative RT-PCR Analysis

For salivary gland expression analysis, 5 pools of 3 glands pairs were isolated per experimental group at 8 and 12 days after dsRNA injection. Saliva was harvested to evaluate the translation silencing by SDS PAGE. The salivary gland tissue was disrupted using a Teflon homogenizer and total RNA was extracted using the RNAqueous kit (Ambion). 100 ng of total RNA was reverse-transcribed using oligo(dT)_15_ (Promega) and Transcriptor reverse transcriptase (Roche). Quantitative real-time PCR was performed in a Roche LightCycler 480, with iQ™ SYBR® Green Supermix (Bio-Rad). For the selection of suitable reference genes, 10 different sets of primers were designed and tested in qPCR. Results were analyzed using the BioGazelle qbase plus 1.5 software revealing that a combination of the *β-tubulin*, *tsetse antigen 5* (*tag5*) and *gapdh* genes provide the best normalization results for the salivary gland tissue. Used primers were: tsetse salivary gland protein 1 (Tsal1) sense (5′-ATGTTCTCTACGCGCCATAC-3′), Tsal1 antisense (5′-GCCAATTAGCACCTGATACC-3′), Tsal2A sense (5′-TTCGGTACTCGATGAGTAGG-3′), Tsal2A antisense (5′-CTGACCAACGTCAAGCTACA-3′), Tsetse antigen 5 (TAg5) sense (5′-GCCAAGCTCAGCTAAGTTCT-3′), TAg5 antisense (5′-GGCCAAGCTGGCTGAATACA-3′), β-tubulin sense (5′-GTAACGACCATGACGTGGAT-3′), β-tubulin antisense (5′-AGAGGTTCGCAACAGTATCG-3′), GAPDH sense (5′-TCGAACACCGACGAATGAGT -3′), GAPDH antisense (5′-AAGAGTGCCACCTACGATGA-3′). The amplicon sizes were respectively 124, 118, 106, 105 and 137 bp. For all primers sets, each PCR cycle consisted of 10 s denaturation at 94°C, 5 s annealing at 60°C and 30 s extension at 72°C. The specificity and efficiency of each primer set was determined by qPCR experiments on cDNA dilution series, melting curve and agarose gel electrophoresis. Gene expression was normalized using *β-tubulin*, *tsetse antigen 5* (*tag5*) and *gapdh* as reference genes using the Biogazelle qbase plus 1.5 software package.

### Gel Electrophoresis and Western Blotting on Tsetse Fly Saliva Proteins

Saliva proteins, salivary fractions or recombinant Tsal1 and Tsal2 were run under reducing and denaturing conditions on a 12% SDS polyacrylamide gel and stained with silver salts or Coomassie Brilliant blue. Western blot analysis was performed by transferring the proteins to nitrocellulose membranes (Hybond C, Amersham) and by specific detection using purified rabbit IgGs against saliva, Tsal1 and Tsal2A and a peroxidase-conjugated anti-rabbit IgG (1/2000, Sigma) as secondary antibody. Saliva proteins were also separated in native conditions to study the high molecular weight (HMW) complexes. Briefly, 10% polyacrylamide gel were run at 4°C using TAE (40 mM Tris, 5 mM sodium acetate, 1 mM EDTA) as running buffer and applying a voltage of 100 V for 3 hours. Protein band visualisation using non-fixing SeeBand solution (GEBA) allowed electro-elution (5 h, 100 V) of the HMW complexes in Laemmli buffer using GebaFlex tubes (GEBA) and subsequent compositional analysis on SDS-PAGE. Densitometric analyses to determine the effects of the Tsal1&2 RNAi were performed as described elsewhere [11]. Protein profiles for 5 pools of 3 glands pairs per experimental group at 8 and 12 days after dsRNA injection were analyzed by separating 5 µg saliva on 12% SDS polyacrylamide gels.

### Gut Protein, Nucleic Acid and Hematin Content Determination

Individual tsetse guts were harvested at eight and twelve days after dsRNA injection and 72 hours after the last blood meal. In addition to the RNAi treated flies, guts of 72 h starved teneral flies were dissected. Entire guts were collected in 150 µl physiological salt solution and homogenized by sonication for 5 seconds. Homogenates were diluted respectively 1∶8 and 1∶40 in physiological salt solution and protein concentrations were quantified using the BCA kit (Pierce). The calculated concentrations were used to determine the total protein content in the individual gut homogenates. 5 µl of the individual gut homogenates was also run on a 2% agarose gel, followed by ethidium bromide staining. Relative integrity of the nucleic acids in the ingested blood meal was analyzed in function of the migration distance. A threshold was set at 300 bp to differentiate between profiles of a normal and perturbed digestion, allowing χ^2^ testing. To determine the relative RNA/DNA composition, gut extracts were treated for 1 h at 37°C by respectively a substrate specific dsDNAse or ssRNAse provided in the Megascript RNAi kit (Ambion) followed by separation and visualisation on a 2% agarose gel.

To determine the hematin contents, the gut homogenates were 1∶1 diluted in 100% formamide and centrifuged for 10′ at 800× *g*. The extracts without the top lipid layer as well as a standard ½ serial dilution of hematin (10 dilutions starting from 250 µg/ml in a 50% formamide solution) was analyzed by measuring the optical density at 405 nm. Hematin contents in the guts were calculated based on the determined concentrations in the extracts.

## Results

### In Silico Analysis of Tsal1 and Tsal2 cDNA Clones

Screening of a λgt11 tsetse salivary gland expression library with serum from saliva-immunized rabbits, lead to the identification of the full-length cDNA sequences encoding two earlier identified secreted proteins with 39% identity and 59% similarity, tsetse salivary proteins 1 and 2 (Tsal1 and 2) [5], [6]. Tsal2 was shown to include two isoforms that differed only by 10 amino acids, Tsal2A and Tsal2B [6]. The different members of the Tsal family have a characteristic NUC domain and share sequence similarity with a number of putative orthologs found in *Culex quinquefasciatus* mosquitoes and New and Old World sand flies (Figure 1A). Homology was also detected with hepatopancreatic enzymes found in crabs, shrimps and prawns, e.g. the *Marsupenaeus japonicus* DNA/RNA non-specific nuclease and the nuclease of the prokaryotic organism *Serratia marcescens* (Figure 1A). Suggestive for a similar secondary structure, the positions of the cysteine residues in all aligned sequences are strikingly conserved (Figure 1A). Moreover, most residues predicted to be important in the active site (Figure 1A, box) for structure determination, substrate and cofactor binding of the archetypical *Serratia marcescens* and the *M. japonicus* nuclease [19], [20] are particularly conserved in Tsal1 and Tsal2 (Figure 1B). However, an equivalent for His^89^ an important basic residue for catalysis in the *S. marcescens* nuclease, is lacking in Tsal1 and Tsal2 (purple residue in Figure 1A, Figure 1B and 1C). Structure prediction within the NUC domain based on experimentally solved nuclease structures suggests a structural homology of both tsetse fly proteins within the putative nuclease active site region (Figure 1C), but the missing His residue casts doubt on their nuclease activity.

### Tsetse Saliva Exerts High Affinity Nucleic Acid Binding and Residual Endonuclease Activity

Total tsetse fly saliva exhibits only residual endonuclease activity as evidenced by a genomic DNA (gDNA) and plasmid DNA (pDNA) digestion assay using migration on an agarose gel and hyperchromicity as read-outs for hydrolytic activity. Confirmation of nuclease activity in saliva that was harvested by induced probing, excluded the possibility that this enzymatic activity resulted from a contamination during the dissection procedure. Nuclease activity was exerted in a broad temperature range with an optimum between 35 and 45°C. Activity could be observed in a broad pH range (pH 3.5–11, Figure 2A). DNase activity was dependent on divalent ions (Mn^2+^ = Co^2^ = Mg^2+^ > Ca^2+^ > Cd^2+^ ≥ Ni^2+^) as cofactors and could be completely inhibited by the addition of EDTA (data not shown). Apparent catalytic rates, determined from hyperchromicity assays in 3 independent experiments at 37°C, were 0.85±0.35 ΔmAU_260 nm_/min×mg at pH 4.0 and 0.25±0.10 ΔmAU_260 nm_/min×mg at pH 7.0. Given that a unit is defined as the amount of nuclease required to obtain a ΔO.D._260 nm_/min of 0.001 ( = 1 mAU) [16], [17], saliva has only residual dsDNAse activity of 0.85 U/mg and 0.25 U/mg under the two respective buffer conditions. No significant catalytic activity was observed when using dsRNA and ssDNA as substrates in different pH conditions. Despite the low rate nuclease activity, total tsetse fly saliva displays a high affinity DNA binding potential in a broad pH range and with an acidic pH optimum. This was determined by surface plasmon resonance (SPR) using a streptavidin sensor chip with immobilized biotinylated DNA or by using a CM5-chip coated with saliva. Binding experiments revealed an apparent high affinity with a very slow dissociation (Figure 2B).

### Tsal Proteins are the Main Constituents of the Active Saliva Fractions

The Tsal proteins constitute approximately 40% of the total proteome of tsetse fly saliva (7). Saliva was separated on a superdex 200 size exclusion column and individual fractions were analyzed for nuclease and DNA binding activity (Figure 3A). Most activity was detected in fractions that correspond to the monomeric Tsals (Figure 3A, II) as well as in the >150 kDa peak fractions (Fig. 3A, I). The dsDNAse activity of the native monomeric Tsal fractions was observed in a broad pH range (pH 5–11). All peak fractions (I, II and III) that exert DNA binding and hydrolytic activity contain the full length Tsal protein or Tsal derived fragments as determined by Tsal-specific immune detection in western blot (Figure 3B). Native gel electrophoresis combined with compositional analysis revealed that saliva consists of 4 high molecular weight complexes of >150 kDa (corresponding to peak I, Figure 3A), that all contained the Tsal proteins (Figure 3C).

### Tsal1 and Tsal2 are DNA/RNA Non-specific Nucleic Acid Binding Proteins

Recombinant Tsal1 and Tsal2A, purified through Ni-NTA affinity and gelfiltration chromatography from the periplasm of *E. coli* (Figure 4A&B), were immobilized onto a CM5 chip for the purpose of SPR analysis. Onto the immobilized Tsal proteins, kinetics of association and dissociation of dsDNA, ssDNA and dsRNA nucleic acid analytes at different concentrations was monitored as response units (Figure 5). The kinetics of association and dissociation reflects the affinity of the different analytes for Tsal1&2. The obtained experimental sensograms were fitted onto a Langmuir 1∶1 binding model with local R_max_, yielding the association and dissociation constants (k_a_ and k_d_) from which the affinity (K_D_) values were calculated for Tsal1 and Tsal2 for each tested analyte. These studies consistently revealed high affinity binding with an acidic pH optimum (pH 4.0) of dsDNA onto both Tsal1 and Tsal2A with apparent K_D_ values of respectively 452 pM and 464 pM for a 161 bp DNA fragment and 1.98 nM and 1.92 nM for a 25 bp A/T/G/C-scrambled DNA fragment (Figure 6, Table 1). PolyA/T dsDNA revealed to bind with similar K_D_ (1.03 nM and 1.30 nM respectively), while polyG/C and dsDNA fragments with purine/pyrimidine repeats [poly(AT) and poly(GC) dsDNA] bound with lower affinities to Tsal1&2 (Figure 6, Table 1). dsRNA (121 bp) was bound with K_D_ values of 3.11 nM and 1.71 nM respectively (Figure 6, Table 1), which was only a slightly lower affinity as for the corresponding dsDNA from which the dsRNA was *in vitro* transcribed. Tsal1 and Tsal2 display higher affinities for double stranded than single stranded analytes, which mainly resulted from faster association (Table 1). Affinities for most mixed single stranded DNA (ssDNA) analytes were slightly above 100 nM for Tsal1 and lower than 100 nM for Tsal2. Both Tsal1 and Tsal2 appeared to display a preference for purine rich ssDNA (polyA and polyG).

### Tsal1 and Tsal2 Exert Residual dsDNAse Activity

Similar as for total saliva, purified recombinant Tsal1 and Tsal2A (Figure 4) exerted only residual dsDNAse activity. Activity was observed in a broad pH range (pH 5.5–11, Figure 7) and highly comparable to what was observed for the native Tsal fractions. Consistent with the activity in total saliva, the Tsal proteins have a substrate preference for dsDNA as no significant dsRNAse and ssDNAse activity could be detected. Preferred co-factors of the individual recombinant proteins were Mn^2+ = ^Co^2^≥ Mg^2+^ > Cd^2+^ ≥ Ni^2+^ (data not shown). Calculated catalytic activities from hyperchromicity experiments conducted at pH 7.0, were in the same low range as for total saliva, respectively 2.7±0.5 and 1.2±0.3 ΔmAU_260 nm_/min×mg for recombinant Tsal1 and Tsal2.

### In vivo Functional Analysis of Tsal1 and Tsal2 by RNA Interference (RNAi)

RNAi was applied for *in vivo* functional analysis of Tsal1 and Tsal2 by intrathorax injection of 15 µg dsRNA per fly. Throughout all performed experiments, a highly selective Tsal1 and Tsal2 silencing with an approximate 90% inhibition at the mRNA level for Tsal1 and a reduction up to 95% for Tsal2 could be achieved by day 12 after dsRNA injection (Figure 8A). The total Tsal protein band intensity was reduced by respectively 23–38% and 48–53% for the Tsal1 and Tsal2 RNAi (Figure 8B&C). This partial Tsal silencing did not impair the overall blood engorgement prior to dissection [GFP RNAi: 17.33±1.11 mg (*n* = 52); Tsal1 RNAi: 21.99±1.90 mg (*n* = 40); Tsal2 RNAi: 21.79±1.53 (*n* = 50)].

Tsal2 silencing reached higher efficiencies than for Tsal1, and resulted in a generally perturbed digestion of nucleic acids, proteins and heme compounds in the blood meal. The degradation of nucleic acids in the tsetse fly alimentary tract was significantly affected (P = 0.00066, Figure 9A). 87% of the detected nucleic acids (Figure 9A), mainly consisting of RNA, originated from the engorged blood meal rather than from the gut cells as was determined by comparing the gut contents of fed and 72 h starved non-fed flies. In the Tsal2 RNAi group, 41% of the flies displayed a perturbed nucleic acid digestion determined at 8 and 12 days p.i. and after 72 h starvation, while in the GFP and Tsal1 RNAi group this was only 3% and 7% respectively. Upon Tsal2 silencing, higher hematin (Figure 9B) and protein contents (Figure 9C) were detected in the intestinal tracts at day 8 (P<0.05) and day 12 after dsRNA injection (P<0.01). Also when hematin and protein contents were calculated relative to the individually ingested blood meal weights, statistically significant differences in ratios were found for Tsal2 as compared to control RNAi treated flies. We tentatively conclude that for the Tsal proteins, a threshold silencing efficiency of 50% (Figure 9D, dotted line) has to be reached before digestive problems can be observed.

## Discussion

A recurrent observation from in-depth transcriptome and proteome analyses in blood feeding arthropods is the putative presence of secreted endonucleases in the salivary glands. This is remarkable as nuclease enzymes are mostly found in the posterior part of the alimentary tract where they are generally produced by pancreatic (vertebrates) or hepatopancreatic cells (invertebrates). So far, the actual presence of nuclease activity in the saliva of blood feeding arthropods had only been documented in the *Culex pipiens quinquefasciatus* mosquito [14].

Here we demonstrate that in the obligate blood feeding tsetse flies, two major salivary components, Tsal1 and Tsal2 [45.6 kDa and 43.9 kDa, [1], [5]], display a significant sequence similarity with the Pfam01223 nucleases, particularly within the active site region (Figure 1). This protein family, characterized by the presence of a DNA/RNA non-specific endonuclease (NUC) domain with a beta-beta-alpha metal finger that coordinates a catalytically important divalent cation, includes nucleases from the shrimp *Marsupenaeus japonicus* [19], the Kamchatka crab (*Paralithodes camtschaticus*) [21], the mosquito *Culex pipiens quinquefasciatus* [14] and the archetypical enzyme from the bacterium *Serratia marcescens* [22], [23]. In addition to the presence of the NUC Smart motif, the highly conserved positions of the cysteine residues in Tsal1&2 as compared to the shrimp nuclease, suggested a similar secondary structure which is strengthened by comparison of Hidden Markov Models and 3D structure prediction. As such, *in silico* analysis identified Tsal1 and Tsal2 as saliva proteins that could interact with nucleic acid substrates.

Homogenous recombinant Tsal1 and Tsal2 proteins were obtained from the periplasm of *E. coli* and were found in surface plasmon resonance (SPR) studies to display high affinity binding properties in a broad pH range and with an acidic pH optimum for dsDNA and dsRNA and a binding at lower affinities for ssDNA. As such, the recombinant Tsal1&2 proteins appear to be DNA/RNA non-specific with a preference for, but not restriction to, duplex nucleic acid analytes. Our SPR studies additionally illustrate a slight preference of both Tsal1 and Tsal2 for purine-rich analytes, as evidenced with polyA and poly G ssDNA. Remarkably, although the binding properties of Tsal1&2 are sugar non-specific and do not exclude single stranded substrates, this study revealed only a very weak catalytic activity that is specific for dsDNA. A similar catalytic preference has been reported for the nucleases of the Kamchatka crab [21] and the *Culex* mosquito [14]. Collectively, these data illustrate that also the Tsal nucleases preferentially cleave dsDNA despite their *in silico* classification as DNA/RNA non-specific nucleases [reviewed in [24]]. Anisimova *et al.* have already highlighted this in a phylogenetic analysis (which included the Tsal1 and Tsal2 sequences) and documented that the duplex-specific members of this enzyme family have a characteristic longer NUC domain (≥ 16 amino acids) than the non-specific members that cluster around the *Serratia* nuclease [25]. Our data indicate that the dsDNA preference results from catalytic selectivity given that binding is not exclusive.

Information concerning the dsDNA hydrolytic mechanism could be mainly deduced from the *S. marcescens* nuclease, where structural information at 1.1 Å is available and site-directed mutagenesis has identified several residues that are crucial for catalytic activity [20], [26], [27], [28]. Although the active site region of *S. marcescens* is located more N-terminal than those predicted for Tsal1, Tsal2 and the shrimp nuclease, nearly all residues that are considered to be required for catalytic activity, cofactor and substrate binding are located at relatively conserved locations. However, Tsal1 and Tsal2 lack an equivalent of the His^89^ residue in *S. marcescens* and the His^211^ residue in *M. japonicus* that is thought to play a crucial role as general base to activate a water molecule as nucleophile in the catalytic reaction [19], [20]. Instead of this histidine residue, the Tsal proteins have a glutamine residue [1], [5]. The same substitution in the *S. marcescens* nuclease (His^89^Gln) was shown to result in an enzyme with an activity of <0.001% as compared to the wild type [20]. This is consistent with the very low remaining activity that we recorded for the Tsal proteins (<3 U/mg). The fact that the CuquEndo contains the corresponding His^89^-residue explains the much higher potency of the mosquito nuclease as compared to the tsetse Tsal proteins. The low nuclease activity of Tsal1&2 is only partially compensated by the relative high abundance of these tsetse salivary nucleases that represent about 40% of the total saliva content. As it is estimated that the *G. m. morsitans* tsetse fly injects around 4 µg saliva proteins during a single feeding event, approximately 1.6 µg of Tsal proteins will be used during the blood meal uptake [6]. Their abundant presence in high molecular weight complexes would also prevent fast dissociation from the feeding site where the nucleic acid binding and/or nuclease activity could be required. Indeed, local cell lysis and the activation of granulocytic cells (e.g. neutrophils) are likely to result in the release of genomic DNA and neutrophil extracellular traps [NETs, [29]] at the feeding site. Several aspects of this extracellular DNA could be relevant for the tsetse feeding physiology, including its viscosity, pro-thrombotic activity [30] and potential cytotoxicity [31], [32]. It is believed that nuclease activity in saliva of hematophagous insects might significantly influence viscosity of the blood meal [14]. Recently, *in vitro* experiments have shown that DNAse can also prevent thrombus formation onto extracellular DNA that serves as a scaffold with pro-coagulant features [30]. As such, salivary nucleases are likely to support the feeding event and blood meal processing by contributing to anti-hemostasis and reduction of the viscosity of the incoming blood. After engorgement, the majority of the salivary proteins has probably been re-ingested from the blood pool and the nuclease activity or the strong DNA binding potential with very low off-rates could represent a protective mechanism for the insect crop or for the epithelial layers that line the insect alimentary tract. Indeed, genomic DNA released by neutrophils was recently documented to contribute to cytotoxicity to endothelial cells [31], [32], a feature called NETosis that can be abrogated by DNAse treatment [32]. The saliva containing blood subsequently moves via the proventriculus into the anterior part of the midgut, undergoes a fast ATP-dependent dehydration [33], [34] and is digested in the tsetse alimentary tract, a process to which the nuclease activity might contribute. Here, the Tsal proteins exert their limited nuclease activity in a pH range that is compatible with the conditions that occur in the tsetse alimentary tract. Collectively, there are several indications from literature that the nuclease activity would be supportive for the blood feeding physiology.

The biological role of the Tsal proteins could be in the digestive tract as well as at the feeding site where saliva proteins contribute to the generation of a local blood pool [3], [8], [9]. Feeding of tsetse flies on immunized mice with high titers of anti-Tsal IgGs did not affect their feeding performance [11]. These observations were corroborated by the high reproductive performance and very low mortality rates of the tsetse fly colony (Institute of Tropical Medicine Antwerp, ITMA) which is maintained by feeding on rabbits that throughout this process raise strong anti-Tsal humoral responses. The absence of a phenotype upon feeding on immunized animals was correlated with the inability of these antibodies to inhibit the nuclease activity of the Tsal proteins (data not shown). Also based on *in vivo* RNA interference, no negative effects of Tsal protein silencing on feeding, determined by quantifying the engorged blood volumes, could be demonstrated. However, in Tsal2 RNAi treated flies, we consistently observed a perturbed processing of the blood meal. Evidence for a generally impaired digestion was provided by higher remaining nucleic acid, hematin and protein contents in the guts of Tsal2 RNAi treated flies. Given that phenotypic effects were only observed in the Tsal2 RNAi group, despite the very similar biochemical properties of Tsal1 and Tsal2, we suggest that a threshold of 50% protein silencing has to be achieved to reveal this phenotype. This would also underscore the importance of the high expression of these proteins, as the remaining Tsal proteins are insufficient to ensure an optimal digestion. The possibility of Tsal1&2 fulfilling a DNA scavenger function was tested by feeding flies with the recombinant Tsal proteins followed by monitoring their perfusion into the insect hemolymph. Although trace amounts of native Tsal could be detected in the hemolymph, no perfusion of recombinant protein from the gut into the circulation could be detected (data not shown).

Collectively, this study characterized Tsal1 and Tsal2 as major components in tsetse saliva with high affinity for binding nucleic acids but with limited dsDNAse activity. Our *in vivo* RNAi experiments indicate that these salivary proteins contribute in part to the processing of the ingested blood meal in the tsetse fly midgut. The documentation of endonuclease activity in *Culex pipiens quinquefasciatus* [14] and the presence of putative endonuclease-encoding transcripts in sandflies *Lutzomyia longipalpis* [13] and *Phlebotomus argentipes* [35] clearly suggest that the presence of this type of enzymes or DNA-binding proteins in the saliva is not limited to the tsetse fly alone, but that they are important in a range of hematophagous insects.
